# Supplementary material for: Interleukin 22 disrupts pancreatic function in newborn mice expressing IL-23
Source: Nat Commun. 2019 Oct 4;10:4517. doi: 10.1038/s41467-019-12540-8 (PMC6778080; doi:10.1038/s41467-019-12540-8)
Supplement: Supplementary file 1 — Supplementary Information [file 41467_2019_12540_MOESM1_ESM.pdf]

Interleukin 22 disrupts pancreatic function in newborn mice expressing IL-23

Lili Chen et al.

Supplementary Figures

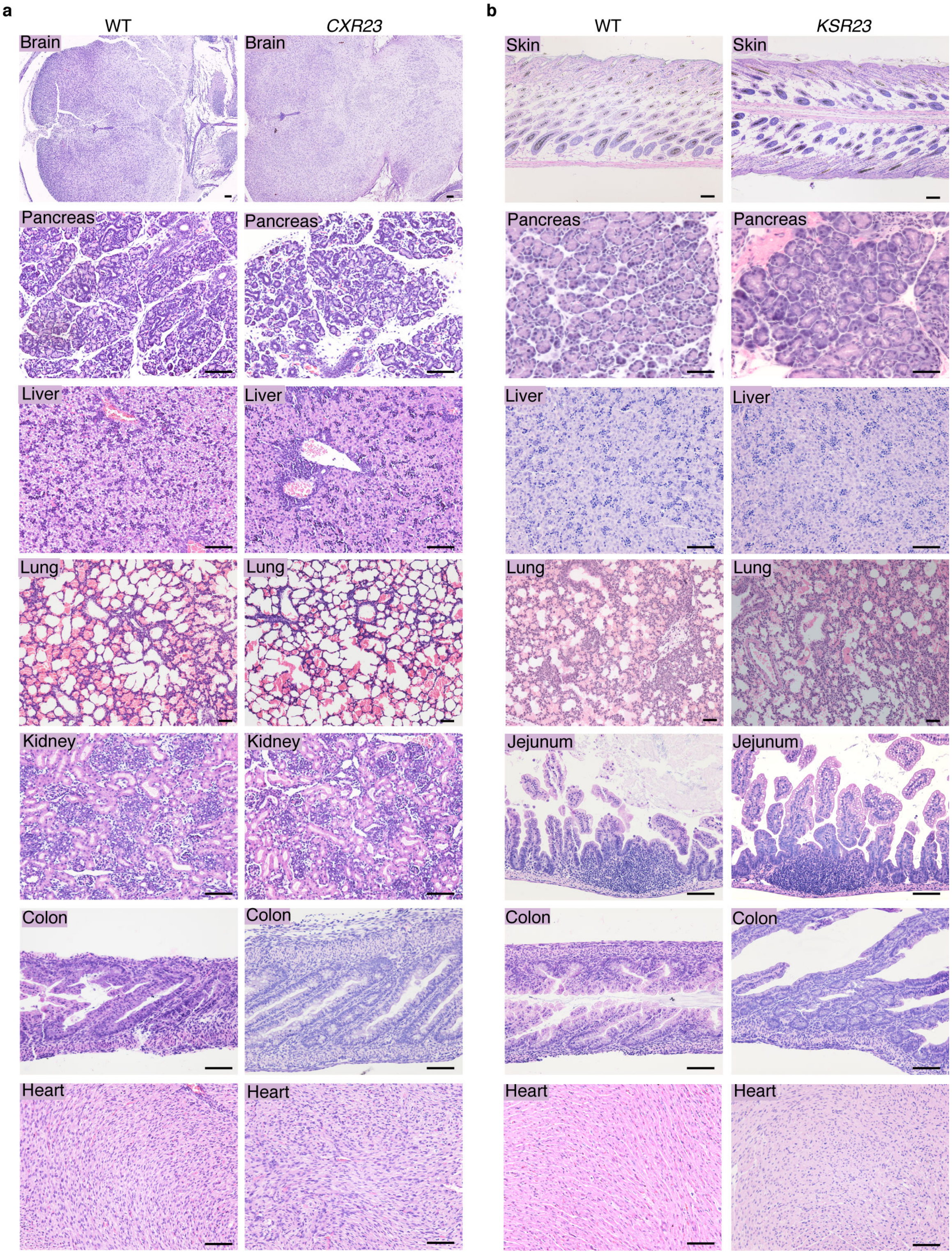

Supplementary Fig. 1 *CXR23* and *KSR23* mice do not develop systemic inflammation. **a-b** Representative H&E section of indicated organs from WT and *CXR23* mice at P1 (**a**) and WT and *KSR23* (**b**) at P5. Scale bars = 50μm.

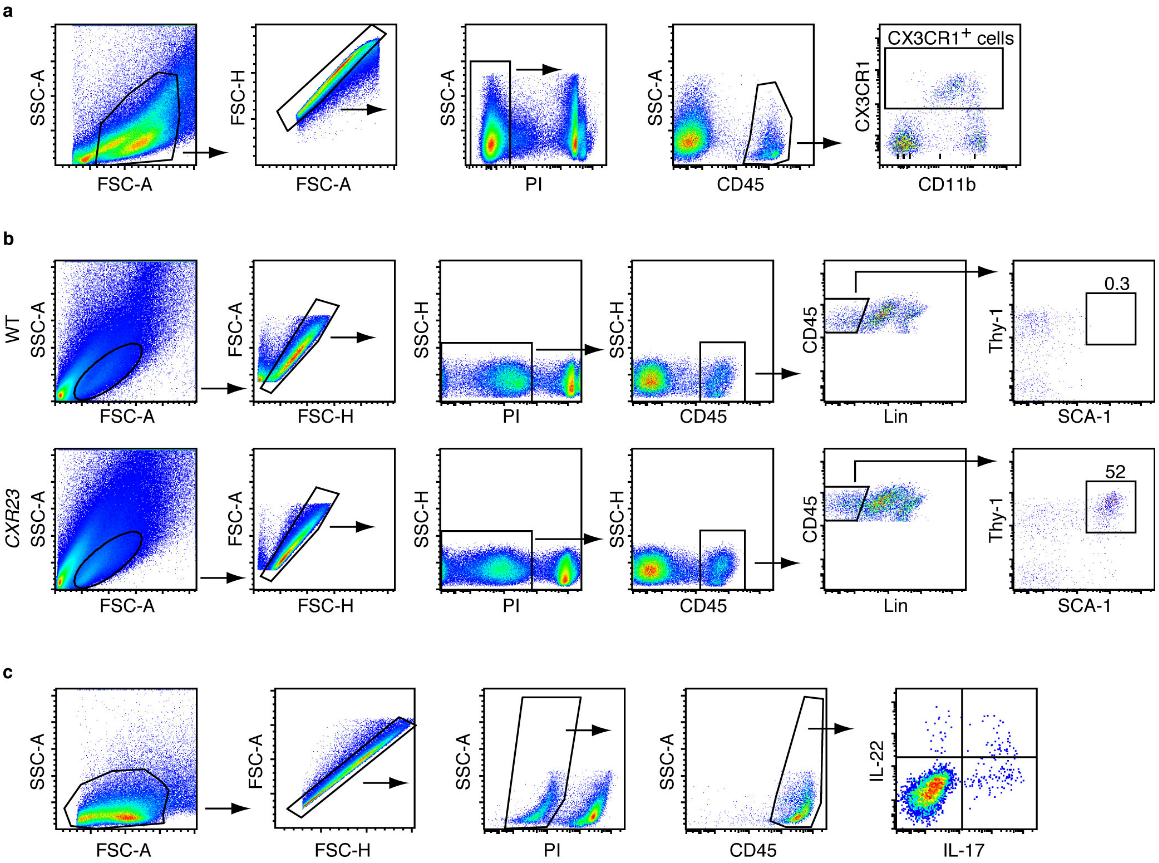

Supplementary Fig. 2 Gating strategies used for flow cytometry. **a** Gating strategy for analysis of CX3CR1$^+$ cells in the intestine (Fig. 1h and 1i). **b** Gating strategy for analysis of ILC3 in the intestine (Fig. k). **c** Gating strategy for analysis of cytokines expression in IL-23 activated CD4$^+$ T cells (Supplementary Fig. 5d).

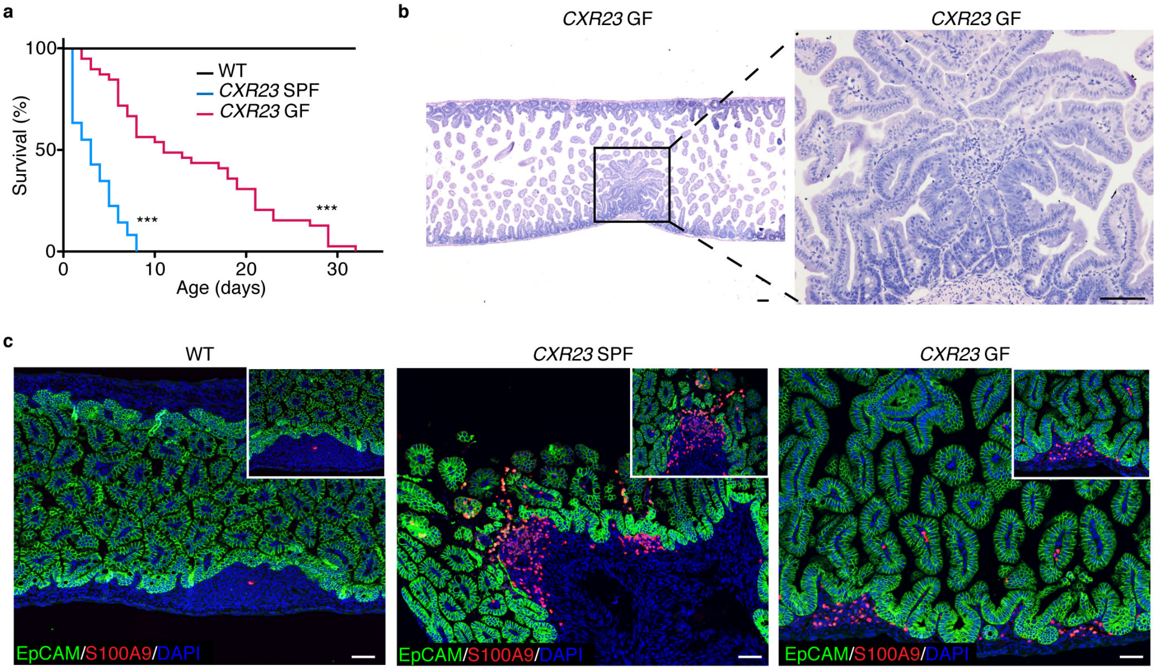

Supplementary Fig. 3 *CXR23* germ-free mice have increased lifespan when compared to *CXR23* SPF mice but die at an early age. **a** Survival curves of WT, *CXR23* SPF and *CXR23* GF mice over time (WT n=25, *CXR23* SPF n=51, *CXR23* GF n=40). **b** Representative H&E section of the small intestine of *CXR23* GF mice at P7. **c** Immunostaining of the small intestine of *CXR23* SPF and *CXR23* GF mice showing the accumulation of S100A9$^+$ neutrophils. Zoomed-in boxed area shows disrupted EpCAM$^+$ intestinal epithelial cells and extravasation of neutrophils into the gut lumen of *CXR23* SPF mice. Neutrophils accumulate in areas contining intact epithelium in *CXR23* GF mice (inset). Scale bars = 50μm. Source data are provided as a Source Data file

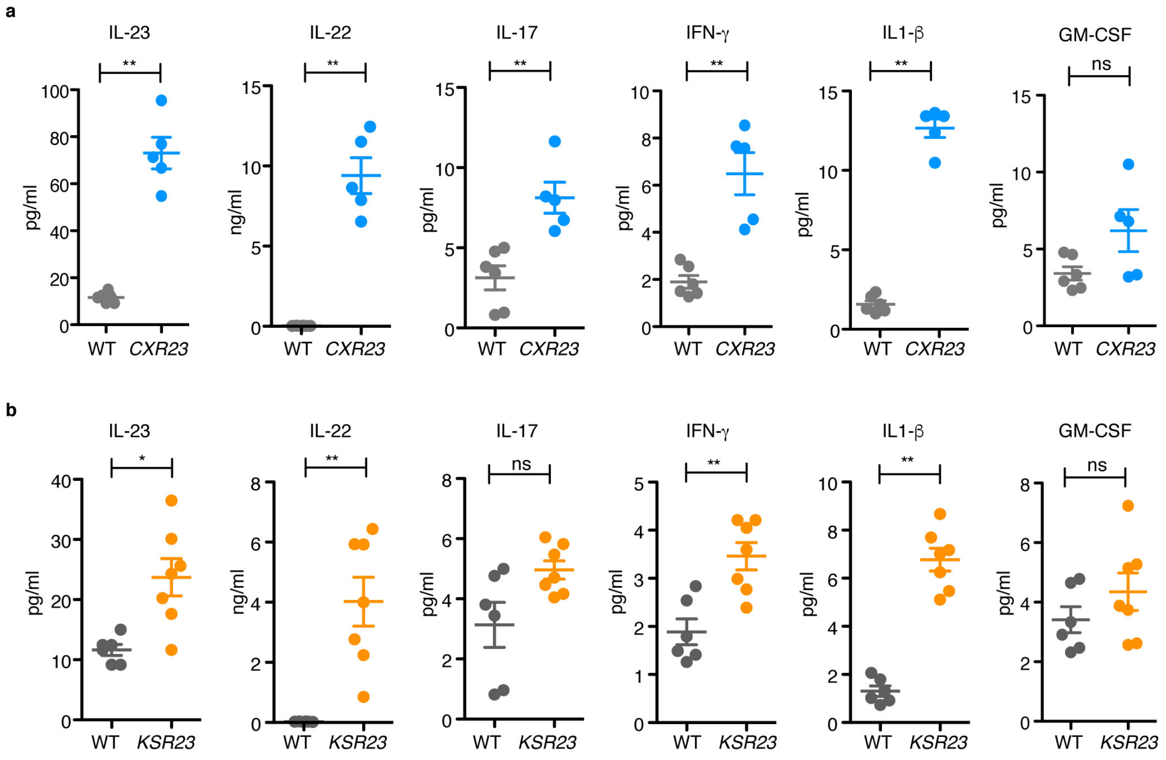

Supplementary Fig. 4 Quantification of cytokines in the serum of WT (P1-P5). **a-b** *CXR23* mice at P1 (**a**) and *KSR23* mice(**b**) at P5 (n =5-8 mice/group). Data are shown as mean ± SEM, statistical analysis by nonparametric Mann-Whitney test. ns $p>0.05$, *$p<0.05$, **$p<0.01$. Source data are provided as a Source Data file

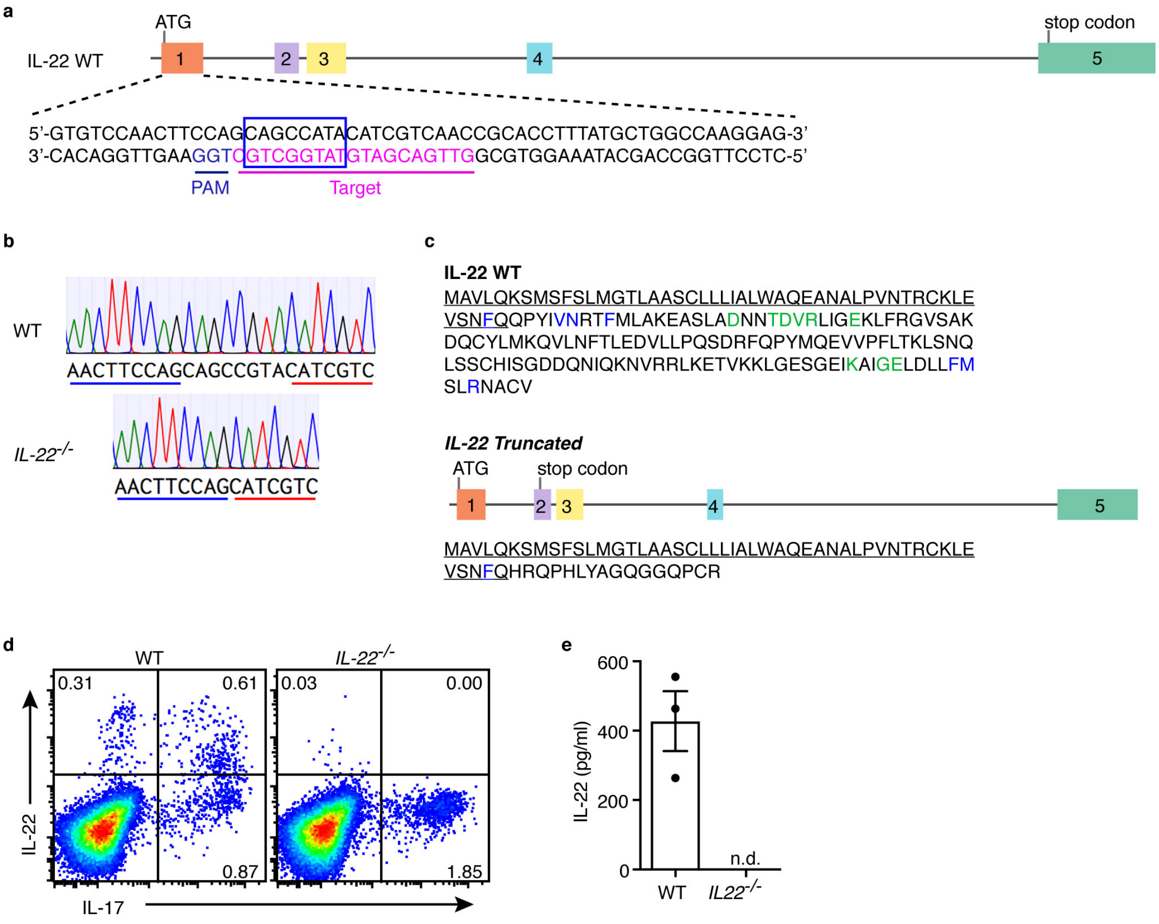

Supplementary Fig. 5. Generation of IL-22-deficient mice by CRISPR/Cas9 technology. **a** Schematic illustration of the IL-22 gene structure and part of exon 1 sequences. The target sequence and PAM domain are indicated in blue and red, respectively. **b** Genomic sequences of IL-22 (exon 1) from the WT and *IL-22*$^{-/-}$ mice. Note a 8bp nucleotide deletion in the *IL-22*$^{-/-}$ mice, which causes frame-shifting and generates a premature stop codon. **c** IL-22 amino acids sequence and truncated protein generated in IL-22 KO mice. Blue and green labeled amino acids are critical for binding its receptor. Note that truncated protein lacked almost all the critical amino acids for binding its receptor. **d** FACS intracellular staining of IL-22 and IL-17 in IL-23 activated CD4^{+} T cells. **e** IL-22 levels measured by ELISA in the supernatant of activated CD4^{+} T cell cultures. n.d., undetectable. n=3 mice per group. Source data are provided as a Source Data file

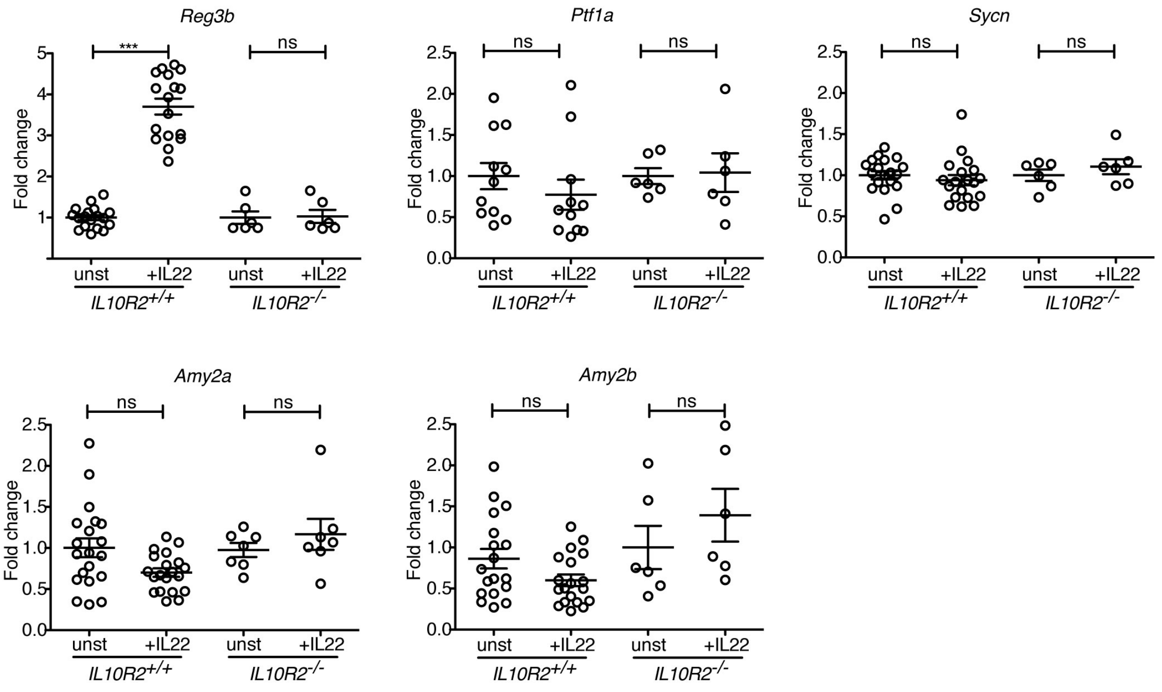

Supplementary Fig. 6 IL-22 induces upregulation of *Reg3β* but does not alter expression of pancreatic enzymes in acinar cell cultures 24h after IL-22 stimulation. Acinar cells from WT and *IL10R2*$^{-/-}$ mice were cultured in the presence of IL-22 recombinant protein. Twenty-four hours after culture, cells were analyzed for the expression of pancreas transcription factor 1a (*Ptf1a*), syncollin (*Sycn*), amylase 2a (*Amy2a*), and amylase 2b (*Amy2b*) by qPCR. The expression of *Reg3β* was significantly increased at 24 h. However, there were no changes in the expression of other pancreatic related genes by IL10R2$^{+/+}$ cells cultured with IL-22. IL-22 did not reduce the expression of pancreatic related gens by IL10R2$^{-/-}$ cells. Data are shown as mean± SEM. n = 6-19 mice/group. Statistical analysis using one-way ANOVA. ns p>0.05, ***p<0.001. Source data are provided as a Source Data file

Supplementary table 1 List of antibodies used in the immunofluorescence studies

Primary antibodies

Description	Catalog #	Vendor	Dilution
Tomato	LS-C340696	LSBio	100
Reg3B	AF5110	R&D Systems	200
Lipase (A-3)	Sc-374612	Santa Cruz Biotech	200
alpha-amylase	ab21156	Abcam	200
Syncollin	sc-390795	Santa Cruz Biotech	200
EpCAM	ab71916	Abcam	200
S100A9	AF2065	R&D Systems	1000
P-Stat3	9145S	Cell Signaling Tech	200

Secondary antibodies

Reactivity	Host	Fluorochrome	Vendor	Catalog#	Dilution
Anti goat IgG	Donkey	Alexa Fluor 594	Invitrogen	A11058	400
Anti goat IgG	Donkey	Alexa Fluor 488	Invitrogen	A11055	400
Anti Rabbit IgG	Goat	Alexa Fluor 488	Invitrogen	A11008	400
Anti Rabbit IgG	Goat	Alexa Fluor 594	Invitrogen	A11037	400
Anti Rabbit IgG	Donkey	Alex Fluor 594	Invitrogen	R37119	400
Anti rat IgG	Goat	Alexa Fluor 594	Invitrogen	A11007	400
Anti rat IgG	Donkey	Alexa Fluor 488	Invitrogen	A21208	400
Anti Mouse IgG2a	Goat	FITC	Abcam	AB97244	400
Anti Mouse IgG2a	Goat	Alexa Fluor 594	Jackson	115-585-206	400
Anti mouse IgG2a	Rat	FITC	ebioscience	11-4210-82	400
Anti Sheep IgG	Donkey	Alexa Fluor 594	Invitrogen	A11016	400

Supplementary table 2 List of primes used in q-PCR studies

Gene	**Forward Primer**	**Reverse Primer**
Ubiquitin	TGGCTATTAATTATTCGGTCTGCAT	GCAAGTGGCTAGAGTGCAGAGTAA
Amy2a	GTGGTCAATGGTCAGCCTTT	CCATCACTGCCAACATTCAC
Amy2b	GGGAGGACTGCTATTGTCCA	CATTGTTGCACCTTGTCACC
Cela1	CAGCCCCATGACTTATCGAG	CTTGTTCCAGTAGGGGTGTG
Ctrl	CCGGTACACAGCACAAGTCT	ACATTGCCCACACCACTGAT
Ctrc	TATAAATGCCCTGCCTGGTC	CCAAGTGTCATCCCTGAGGT
Cpa1	GGGACCCTCGTCAGTGTTTA	GCAGAGATTCGGAGAACCTG
Cpa2	ACAGTCCATGTCCGAGTTCC	CAGCCACGAGGTTATCCATT
Il-23p19	CCAGCAGCTCTCTCGGAATC	GATTCATATGTCCCGCTGGTG
Il-23p40	ACAGCACCAGCTTCTTCATCAG	TCTTCAAAGGCTTCATCTGCAA
Prss2	ACAAATACCGCATCCAAGTG	TGGCAGAATCAACAAACTGC
Ptf1a	GGATCACTCACAAAGCGTCA	TAGCTGGTGGCTGAGGAACT
Pnlip	AGCCATTGGAAGGATCACAG	CGTCGATGTCAACAATTTGG
Pga5	GGAAGAACCTGGCATTTTCA	CACTCCACCTAGCATCAGCA
Reg3b	TGCCTTGTTTCAGATACCACAG	ACCATGGAGGACAAGAATGAAG
Sync	CCAGGTACTGACCTGCCCTA	GCAGTAGAGGGCAGAGATGG
Il22	GCCTTGTTTCAGATACCACAG	ACCATGGAGGACAAGAATGAAG
**PRIMERS USED IN THE NEC EXPERIMENTS**		
Rplo	GGCGACCTGGAAGTCCAACT	CCATCAGCACCACAGCCTTC
Il-23p19	TGGAGCAACTTCACACCTCC	GGCAGCTATGGCCAAAAAGG
Reg3b	TGCCTTGTTTCAGATACCACAG	ACCATGGAGGACAAGAATGAAG
Il22	CGACCAGAACATCCAGAAGAA	GAGACATAAACAGCAGGTCCA
