# Supplementary material for: Interleukin 22 disrupts pancreatic function in newborn mice expressing IL-23
Source: Nat Commun. 2019 Oct 4;10:4517. doi: 10.1038/s41467-019-12540-8 (PMC6778080; doi:10.1038/s41467-019-12540-8)
Supplement: Supplementary file 3 — Reporting Summary [file 41467_2019_12540_MOESM3_ESM.pdf]

nature research

Corresponding author(s): Sergio A. Lira

Last updated by author(s): Sep 11, 2019

Reporting Summary

Nature Research wishes to improve the reproducibility of the work that we publish. This form provides structure for consistency and transparency in reporting. For further information on Nature Research policies, see Authors & Referees and the Editorial Policy Checklist.

Statistics

For all statistical analyses, confirm that the following items are present in the figure legend, table legend, main text, or Methods section.

n/a	Confirmed	
☐	☒	The exact sample size (*n*) for each experimental group/condition, given as a discrete number and unit of measurement
☐	☒	A statement on whether measurements were taken from distinct samples or whether the same sample was measured repeatedly
☐	☒	The statistical test(s) used AND whether they are one- or two-sided<br>*Only common tests should be described solely by name; describe more complex techniques in the Methods section.*
☐	☒	A description of all covariates tested
☐	☒	A description of any assumptions or corrections, such as tests of normality and adjustment for multiple comparisons
☐	☒	A full description of the statistical parameters including central tendency (e.g. means) or other basic estimates (e.g. regression coefficient) AND variation (e.g. standard deviation) or associated estimates of uncertainty (e.g. confidence intervals)
☒	☐	For null hypothesis testing, the test statistic (e.g. *F*, *t*, *r*) with confidence intervals, effect sizes, degrees of freedom and *P* value noted<br>*Give P values as exact values whenever suitable.*
☒	☐	For Bayesian analysis, information on the choice of priors and Markov chain Monte Carlo settings
☒	☐	For hierarchical and complex designs, identification of the appropriate level for tests and full reporting of outcomes
☒	☐	Estimates of effect sizes (e.g. Cohen's *d*, Pearson's *r*), indicating how they were calculated

Our web collection on statistics for biologists contains articles on many of the points above.

Software and code

Policy information about availability of computer code

Data collection	The flow cytometric data: BD LSR II or BD Canto<br>The RNA-seq data: Illumina HiSeq 4000
Data analysis	Statistical analyses : GraphPad Prism 7 software<br>Flow cytometry : FlowJo 10.0<br>RNA-seq: Bioconductor EdgeR package version 3.10.2 Bioconductor/R

For manuscripts utilizing custom algorithms or software that are central to the research but not yet described in published literature, software must be made available to editors/reviewers. We strongly encourage code deposition in a community repository (e.g. GitHub). See the Nature Research guidelines for submitting code & software for further information.

Data

Policy information about availability of data

All manuscripts must include a data availability statement. This statement should provide the following information, where applicable:

- Accession codes, unique identifiers, or web links for publicly available datasets
- A list of figures that have associated raw data
- A description of any restrictions on data availability

All RNA-seq data generated are available at Gene Expression Omnibus (GEO) under the accession number GSE137415 . The authors declare that the data that support the findings of this study are available within the paper and its supplementary information files. Extra data or information are available from the corresponding authors upon request. The raw data underlying Figs. 1,2,3,4,5,7,8,9 and Supplementary Figs. 3–6 are available as Source Data.

Field-specific reporting

Please select the one below that is the best fit for your research. If you are not sure, read the appropriate sections before making your selection.

☒ Life sciences ☐ Behavioural & social sciences ☐ Ecological, evolutionary & environmental sciences

For a reference copy of the document with all sections, see nature.com/documents/nr-reporting-summary-flat.pdf

Life sciences study design

All studies must disclose on these points even when the disclosure is negative.

Sample size	No statistical method was used to predetermine sample size. We described the numbers of animals/samples for each experiments in the figure legends.
Data exclusions	No data was excluded from analysis.
Replication	The number of experiments performed for each panel is indicated in Figure legend.
Randomization	We usually used littermate mice in our experimental and control group.
Blinding	The evaluation of histological disease score was performed in a blinded fashion.

Reporting for specific materials, systems and methods

We require information from authors about some types of materials, experimental systems and methods used in many studies. Here, indicate whether each material, system or method listed is relevant to your study. If you are not sure if a list item applies to your research, read the appropriate section before selecting a response.

Materials & experimental systems

n/a	Involved in the study	
☐	☒	Antibodies
☒	☐	Eukaryotic cell lines
☒	☐	Palaeontology
☐	☒	Animals and other organisms
☒	☐	Human research participants
☒	☐	Clinical data

Methods

n/a	Involved in the study	
☒	☐	ChIP-seq
☐	☒	Flow cytometry
☒	☐	MRI-based neuroimaging

Antibodies

Antibodies used	All antibodies used are listed in Supplementary Table (Source and Catalog number)
Validation	All antibodies against surface-expressed markers used in this study have been previously validated by the manufacturer, as stated on their associated product websites, and by our own lab in previous experiments.

Animals and other organisms

Policy information about studies involving animals; ARRIVE guidelines recommended for reporting animal research

Laboratory animals	C57BL/6 (CN 000664), CX3CR1-cre13 (CN 025524), hK14-cre (CN 018964), and IL10R2KO (CN 005027) mice were purchased from The Jackson laboratory. R23 and IL-22ko mice generated in house, and in C57BL/6 background. IL-22 tdTomato mice were provided by Dr. Scott Durham (NIH, Bethesda, MD).
Wild animals	No wild animal was used in this study.
Field-collected samples	No field-collected sample was used in this study.
Ethics oversight	All animal experiments in this study were approved by the Institutional Animal Care and Use Committee of Icahn School of Medicine at Mount Sinai, and were performed in accordance with the approved guidelines for animal experimentation at the Icahn School of Medicine at Mount Sinai. For the study of induction of necrotizing enterocolitis in mouse, experiments and procedures were approved by the Johns Hopkins University Animal Care and Use committees in accordance to the Guide for the Care and Use of Laboratory Animals.

Note that full information on the approval of the study protocol must also be provided in the manuscript.

Flow Cytometry

Plots

Confirm that:

- [x] The axis labels state the marker and fluorochrome used (e.g. CD4-FITC).
- [x] The axis scales are clearly visible. Include numbers along axes only for bottom left plot of group (a 'group' is an analysis of identical markers).
- [x] All plots are contour plots with outliers or pseudocolor plots.
- [x] A numerical value for number of cells or percentage (with statistics) is provided.

Methodology

Sample preparation	Sample preparation is detailed in methods.
Instrument	The flow cytometric data: BD LSR II or BD Canto<br>The RNA-seq data: Illumina HiSeq 4000
Software	The flow cytometer used to collect the data was BD FACSDiva, and the final analysis was completed using FlowJo (TreeStar).
Cell population abundance	N/A
Gating strategy	We usually gated the lymphocytes based on the FSC-A and SSC-A. Singlets were gated according to the pattern of FSC-H vs FSC-A. The specific cell population was gated on the indicated surface markers as described in the manuscript.

- [x] Tick this box to confirm that a figure exemplifying the gating strategy is provided in the Supplementary Information.
